# Association between Inflammation and Thrombotic Pathway Link with Pathogenesis of Depression and Anxiety in SLE Patients

**DOI:** 10.3390/biom13030567

**Published:** 2023-03-20

**Authors:** Liliana Duca, Nadinne Roman, Andreea Teodorescu, Petru Ifteni

**Affiliations:** 1 Faculty of Medicine, Transilvania University of Braşov, 500036 Braşov, Romania; 2 Department of Clinical Immunology, County Emergency Clinic Hospital, 500326 Braşov, Romania; 3 Clinical Hospital of Psychiatry and Neurology of Brasov, 500123 Braşov, Romania

**Keywords:** systemic lupus erythematosus, depression, anxiety, inflammation, thrombotic

## Abstract

As a complication of systemic lupus erythematosus (SLE), the neuropsychiatric form may manifest with neurological and psychiatric symptoms. Diagnosing neuropsychiatric SLE can be challenging due to the heterogeneity of this disease manifestation and the possibilities of investigation. This research aims to identify the possible associations between inflammation and thrombotic biomarkers alongside anxiety and/or depression manifestations in SLE patients. A group of 65 outpatients were investigated regarding the levels of depression, anxiety, disability, quality of life and other specific serum biomarkers linked with inflammation or coagulopathies. The results showed severe depression in eight participants, moderate depression in 22 (33.85%), and 26 (40%) subjects with mild depression. Anxiety was more prevalent within 64 participants (98.46%), while a degree of disability was reported by 52 participants (80%). Quality of life evaluated by EQ5D revealed a medium value of 1.57, and EQ5D VAS health medium value was 57.95 and was correlated with anxiety. A strong positive correlation between depression, anxiety and antibodies associated with anti-cardiolipin and anti beta2 glycoprotein I antibodies, lupus anticoagulant, ICAM-1, low C4 a and anti-ribosomal P antibodies were identified. These data results suggest that autoimmune/inflammatory and ischemic/thrombotic pathways could contribute to depression and anxiety as neuropsychiatric SLE manifestations.

## 1. Introduction

Systemic lupus erythematosus (SLE) is an autoimmune disease with systemic implications that can affect multiple organisms. Psychiatric manifestations represent a complication of this condition, a phenomenon known as neuropsychiatric systemic lupus erythematosus (NPSLE) directly related to SLE [1]. NPSLE could be the singular or initial manifestation of SLE, and in roughly 30% of individuals with neuropsychiatric symptoms, SLE is the direct cause. NPSLE usually embodies when SLE manifests clinically, and the biomarkers are serologically active. Thus, by identifying the characteristic elements from the clinical evaluation, with the results of serological analyzes and imaging investigations, it can be determined whether the patient has active NPSE or the symptoms are determined by other causes [2].

In 2012, the American College of Rheumatology (ACR) classification criteria for SLE patients were re-examined. The elements were corroborated to enhance clinical importance, meet rigorous methodology demands, and approach new acquaintances regarding the immune response in SLE patients. The neuropsychiatric symptomatology of patients with SLE includes several types of somatic manifestations. Concerning the central motor neuron, cerebrovascular diseases (including stroke), aseptic meningitis, seizures, delirium and dementia, psychoses, mild cognitive impairment, demyelination syndromes, and migraines can be encountered. Regarding the peripheral motor neuron, autonomic and peripheral neuropathies, sensorial-neural hearing loss and myasthenia gravis were identified [3,4]. According to previous data, approximately 50–60% of NPSLE manifestations arise at the beginning of SLE or within the first year of diagnosis, frequently when the disease activity is generalized [5]. At the same time, another study shows contrasting information, suggesting that common NPSLE manifestation are not associated with the disease’s increased activity or severity [6,7]. The identification of the neuropsychiatric form in patients with SLE often represents a challenge for rheumatologists due to the heterogeneity of the clinical picture, the lack of specificity and sensitivity of biological biomarkers, but also the lack of the existence of other well-defined criteria for the accurate establishment of the diagnosis and the orientation toward an efficient care and management plan of patients with SLE [2].

As psychiatric manifestations, anxiety and depression are considered SLE comorbidities and can develop at different stages of the disease, with high variability in the prevalence in different studies, ranging from 8.7–78.6% and 1.1–71.4%, respectively [8]. Depression and anxiety have a high prevalence and lead to a profound drop in quality of life in patients with SLE, and are also essential negative predictors, in the same way as NPSLE decreases survival rates of SLE patients [9,10].

The complexity and heterogeneity of the clinical picture of NPSLE suggest that many factors might trigger neuropsychiatric manifestations. Since the clinical manifestations of NPSLE are varied and it is not established yet if the neuropsychiatric manifestations of SLE are primary or secondary, recent research focuses on identifying other mechanisms involved in the NPSE manifestation. Autoantibodies involved in the neuroimmune response, anti-phospholipid antibodies, complement activity, and cell-mediated and cytokine-mediated inflammation are investigated for associations with NPSLE manifestations [11]. Thus, molecular elements associated with coagulopathy which may cause a neurodegenerative response, are also investigated, in addition to the inflammatory autoimmune response. Still, essential elements are also identified that intervene in the blood barrier of the central nervous system, affecting the permeability and allowing the penetration of specific autoantibodies and immune cells in the central nervous system with mediated neuronal damage [1]. Two paths of pathological mechanisms that may contribute to SLE’s neuropsychiatric manifestations have been identified and addressed in the specialized literature [12]. One of these two main ways of pathological manifestation of NPSLE is related to the vascular dysfunctions manifested at the central nervous system level. In brain tissue of NPSLE, microthrombi, micro and macro-infarcts, and vasculitis were identified as more frequent than in subjects with SLE, suggesting that vasculopathy and coagulopathy are essential factors involved in NPSLE manifestations [13]. Other essential pathological mechanisms involved in the neuropsychiatric manifestations of SLE are linked with the inflammatory and autoimmune response involving specific autoantibodies and inflammation-mediated cellular response. Thus, it is suggested that the phenomenon of accelerated atherosclerosis, together with the deposition of the immune complex and the presence of immune-mediated vascular lesions, interact in these two pathways [14,15].

The complex manifestation of NPSLE is closely related to the disease’s activity. The more the disease manifests an increased activity, the more it seems both immune response mechanisms (inflammatory and vascular) are involved and altered. In addition to the increased manifestation of NPSLE, anti-ribosomal P autoantibodies (Anti RIB P) have been identified as an increased risk factor and poor prognosis for individuals with NPSLE [10]. Brain histology for NPSLE patients suggested that microglia activation might contribute to disease development by affecting the neuronal and synaptic structure and function. Previous research on brain tissue suggests that the presence of cytokines, antibodies and infiltrating cells, by passing through the blood-brain barrier, can cause diffuse injuries [12]. Cytokines can affect neuronal and endothelial tissue through cell death; these dysfunctions were associated with depression, lethargy and increased seclusion [16,17].

SLE has been associated with 116 antibodies, and NPSLE has been associated with the presence of 20 autoantibodies, of which eleven are related to the central nervous system. Still, the neuropsychiatric manifestations have not been fully elucidated or associated with a specific autoantibody [1].

Vascular inflammation presents specific biomarkers through cell adhesion molecules, binding leukocytes with endothelial cells and the extracellular matrix. P-selectin is an essential biomarker in this process of vascular inflammation. It is expressed both at the level of endothelial cells and the level of platelets, influencing the phenomenon of coagulation and thrombi formation during the autoimmune response [18]. In patients with SLE, an up-regulation of P- and E-selectin was found on microparticles and in their soluble forms that correlated with disease activity [19].

The modality of the autoimmune response in patients with SLE manifesting with increased production of P-selectin protein is not fully identified [20] Norwalk. Recent research suggests that through the increased expression of platelets (including P-selectin), a continuous platelet modification response can result in patients with SLE by changing the shape and plasma membrane [21].

Plasminogen activator inhibitor-1 (PAI-1) and intercellular adhesion molecule- ICAM-1 (cell surface glycoprotein) indicate the autoimmune response in SLE. A recent and extensive meta-analysis on ICAM-1 in SLE patients discovered that both blood and urine ICAM-1 concentrations were higher than in SLE patients than control groups. However, no data was explicitly linked to NPSLE [22]. More recent research suggested that an altered fibrinolysis process contributes to a hypercoagulability condition and micro thrombotic events in the Chinese pediatric cohort with SLE, as proved by high levels of PAI-1 and low levels of tissue plasminogen activator [23]. Additionally, recent research results suggest that inflammatory markers are increased in depression, and the extrinsic coagulation pathway is closely related to depression [2]. However, systemic inflammation is linked with depression. It is mainly conjunct with somatic or neurovegetative manifestations such as fatigue, altered sleep, and appetite, as well as depressed mood and anhedonia [24].

Severe psychiatric disorders such as schizophrenia or major depression are associated with an increased risk of thrombotic phenomena and cardiovascular damage [25]. Thus, there are indications that hemostatic phenomena are involved in several types of psychiatric disorders, including mental stress [26]. Although Lupus Anticoagulant (LA) antibodies are not specific for SLE, these types of immunoglobulins can be found in various autoimmune disorders associated with coagulation dysfunctions. Furthermore, LA is associated with other neurological conditions (like stroke and epilepsy), but the LA antibodies are also found in 25% of SLE patients [27,28,29].

Another essential biomarker in neuropsychiatric manifestations of NPSLE is related to Anti RIB P which can be found frequently among patients with SLE [30,31].

Since many factors can influence the immune response of the body to SLE and the presence of neuropsychiatric manifestation is not fully understood from a pathophysiological perspective, our research aims to identify the connection between depression and anxiety and biomarkers possibly associated with NPSL for thrombosis antiphospholipid antibodies, PAI-1, P-selectin, ICAM 1 and among Anti RIB P antibodies.

## 2. Materials and Methods

Cross-sectional research was performed on 65 adult patients diagnosed with SLE according to Systemic Lupus International Collaborating Clinics (SLICC) or ACR criteria at least 6 months before enrollment [3,32]. The participants were investigated between June 2019 and January 2020. The research was conducted in the Department of Clinical Immunology, Brasov County Emergency Clinical Hospital, Romania. Written informed consent was obtained from each participant after the local ethics committee approved the research. All procedures were performed according to local regulations.

No patients were diagnosed with NPSLE at the begging of the research. The exclusion criteria were: history of substance abuse, alcohol abuse, personality disorders, or other major psychiatric diseases.

The subjects were examined clinically and paraclinical, including a complete physical examination, a biological examination with serological determinations for SLE, including high sensitive C-reactive protein (hs CRP), anti-Smith antibody (Anti SM), anti-nuclear antibodies (ANA), Anti-double-stranded deoxyribonucleic acid antibodies (DNA DC), autoantibodies to beta(2)-glycoprotein 1 (ANTI B2 GP1), anti-cardiolipin antibodies (ACL SCR), complement C3 and C4, LA, P-selectin, ICAM 1, PAI 1, Anti RIB P and D-dimers.

Test were performed with different methods. The normal range and the techniques used are depicted in Table 1.

To identify the SLE status, the British Isles Disease Activity Group Index 2004 (BILAG Index) and the Systemic Lupus Erythematosus Activity Index (SELENA-SLEDAI) were used. Only patients without disease activity were included.

To assess the level of disability, the World Health Organization Disability Assessment Schedule (WHODAS) 2.0 was used, and for the quality of life, the European Quality of Life Five Dimension (EQ-5D) tool was used [33,34]. The Hamilton Anxiety Scale (HAM A) was used to assess anxiety, and the Hamilton Depression Rating Scale (HAM D) was used to assess depression. To identify the levels of anxiety and depression, the HAM A and HAM D, applied by a psychiatrist, were used. The cut-off values for depression were below 17 points considered as mild depression, between 18 and 25 as moderate depression, and a score above 26 indicating severe depression. In the evaluation of the anxiety levels, the cut-off values below seven were reported as no anxiety, between 8 and 14 as mild anxiety, between 15 and 23 as moderate anxiety and above the value 24 as severe anxiety, while scores greater than 30 considered as very severe anxiety [35,36].

All collected data were analyzed using IBM Statistical Package SPSS version 20.0 (IBM Corp. Released 2011. IBM SPSS Statistics for Windows, Version 20.0, Armonk, NY, USA). Linear regression was used for detecting possible risk factors associations of antiphospholipid antibodies and inflammation markers for depression and anxiety in SLE patients, while binary logistic regression was performed for dichotomous variables. For a further understanding of the antibodies involved in SLE association with anxiety, depression and quality of life, based on regression analysis results, we performed an Mann Whitney test by comparing groups positive LA, positive, anti-RIB P, positive DNA DC, and Anti SM. To identify differences of with PAI 1, hs CRP, C4 intervals, age and time since disease two-way ANOVA was used. Pearson correlation was performed to identify possible correlation between analyzed variables. Both the correlation coefficients and the p-values were calculated according to a default 95% confidence interval. The significance level was set at p-values less or equal to 0.05.

## 3. Results

The studied population included 65 Caucasian Lupus, whose disease was controlled on background therapy. The participants characteristics are depicted in Table 2. Fifteen patients (23.07%) were smokers.

Highly sensitive C-reactive protein had low values in all patients, and the same was PAI-1 levels. One patient (1.53%) had positive test for P-selectin and 20 patients (30.77%) had high results for ICAM -1. D-dimers positive values were found in 10 subjects (15.38%). Anti RIB P was found in 28 (43.08%) subjects. Fifty-two subjects (80%) were positive for ANA antibodies, while 33 (50.77%) subjects were found with positive Ant-Ro antibodies. Anti-SM antibodies were identified with positive values in two (3.08%) participants. For ANTI B2 GP1, a total number 31 (47.69%) participants were identified as positive. ACL SCR serum levels (screen for IgG and IgM) were present in 36 (55.38%) patients, 31 (47.69%) patients were positive for anti beta2 glycoprotein 1 (screen for IgG and IgM), while LA was present in 31 (47.69%) subjects.

**Figure 1:**
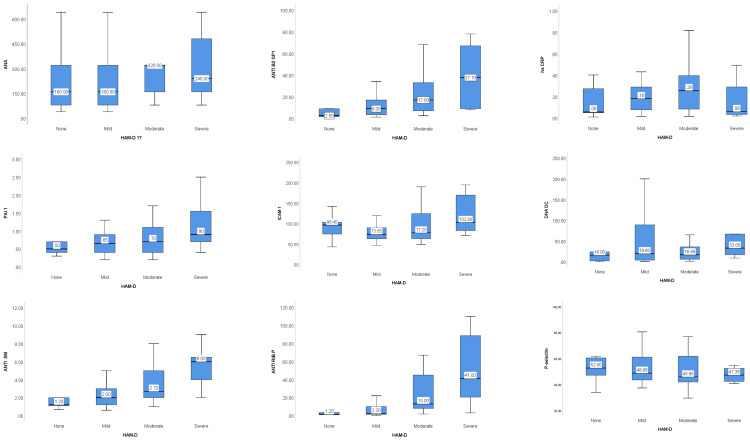

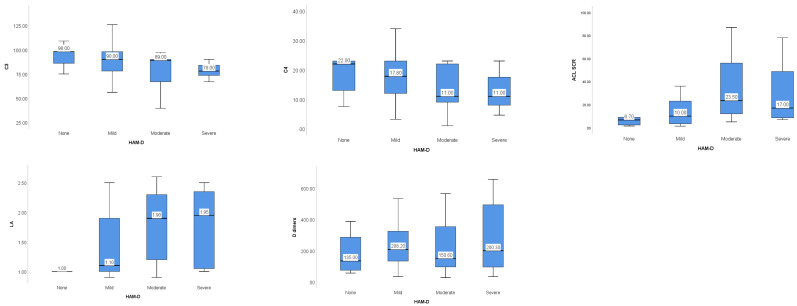
Biomarkers levels graphic representation based on depression categories (HAM D).

Depression was present in 56 (86.15%) patients, 8 (12.30%) subjects had severe depression, 22 patients (33.85%) presented moderate symptoms and in 26 (40%) patients depression was mild. The values of the analyzed biomarkers reported to the levels of depression identified in the number of patients (n = 56) are shown in Figure 1. From the boxplots depicted in Figure 1, the results suggest that most biomarkers serum levels increase alongside depression severity, except for C3, C4 and D-dimers. The values recorded for Antti B2 GP1, PAI 1, Anti SM, Anti RIB P and LA seem to increase as the severity of depression increases.

High sensitive C-reactive protein—hs CRP; anti-Smith antibody—anti SM; anti-nuclear antibodies—ANA; autoantibodies to beta (2)-glycoprotein 1—ANTI B2 GP1; anti-double-stranded deoxyribonucleic acid antibodies—DNA DC; anti-cardiolipin antibodies—ACL; SCR; complement C3 and C4; lupus anticoagulant—LA; P-selectin; intercellular adhesion molecule—ICAM 1; plasminogen activator inhibitor—PAI 1; anti-ribosomal P antibodies—anti RIB P.

**Figure 2:**
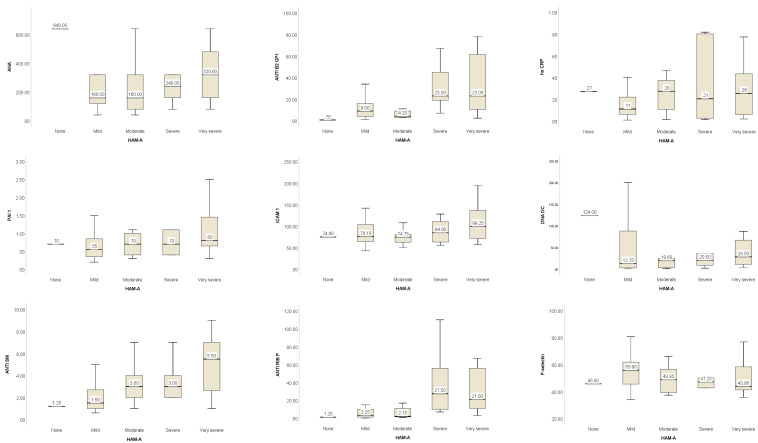

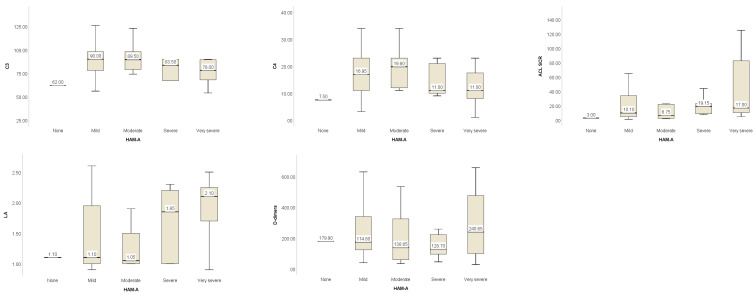
Biomarkers levels graphic representation based on anxiety categories (HAM A).

Anxiety was more prevalent, 64 (98.46%) patients presented anxiety, among these, 16 (24.62%) patients reported very severe anxiety, 6 (9.23%) subjects reported severe anxiety, 10 (15.38%) declared moderate anxiety, and 32 (49.23%) patients were recorded with mild anxiety. The values of the analyzed biomarkers reported to the levels of anxiety identified in the number of patients (n = 64) are shown in Figure 2. The graphic representation between anxiety levels of our sample of SLE patients and analyzed biomarkers suggest that ANA, Anti B2 GP1, ICAM 1, Anti RIB P and LA values increase as the anxiety level becomes severe or very severe. At the same time, P-selectin serum levels seem to be increased in subjects with mild anxiety.

High sensitive C-reactive protein—hs CRP; anti-Smith Antibody—Anti SM; anti-nuclear antibodies—ANA; autoantibodies to beta (2)-glycoprotein 1—ANTI B2 GP1); anti-double-stranded deoxyribonucleic acid antibodies—DNA DC; anti-cardiolipin antibodies—ACL; SCR; complement C3 and C4; lupus anticoagulant—LA; P-selectin; intercellular adhesion molecule—ICAM 1; plasminogen activator inhibitor—PAI 1; anti-ribosomal P antibodies—Anti RIB P. The results of Kruskal–Wallis and multiple comparison results suggest that the values for ANTI B2 GP 1 increase (statistically significant) as the levels of depression increase, from none, to moderate and severe. Regarding ANTI SM autoantibody, the results showed it increased from subjects without depression to those with severe depression and from mild to severe depression. The ANTI RIB P serum values increase as the depression increase from none to moderate and severe and from mild to severe disorder. While the ACL SCR autoantibodies level increased from none to moderate depression and mild to moderate depression. The LA level increased for SLE subjects without depression to subjects with moderate depression. For patients with severe anxiety vs. moderate, the serum levels of ANTI B2 GP 1 were significantly increased; the serum levels for ANTI SM autoantibodies levels between mild and very severe anxiety groups were also increased significantly. At the same time, the level of ANTI RIB P increased significantly from moderate to severe anxiety and from mild to severe anxiety.

A degree of disability was reported by 52 (80%) patients, 6 (9.23%) patients had moderate disability and 46 (70.77%) reported mild disability.

Quality of life evaluated by EQ5D revealed a medium value 1.57.

### Correlations

**Figure 3:**
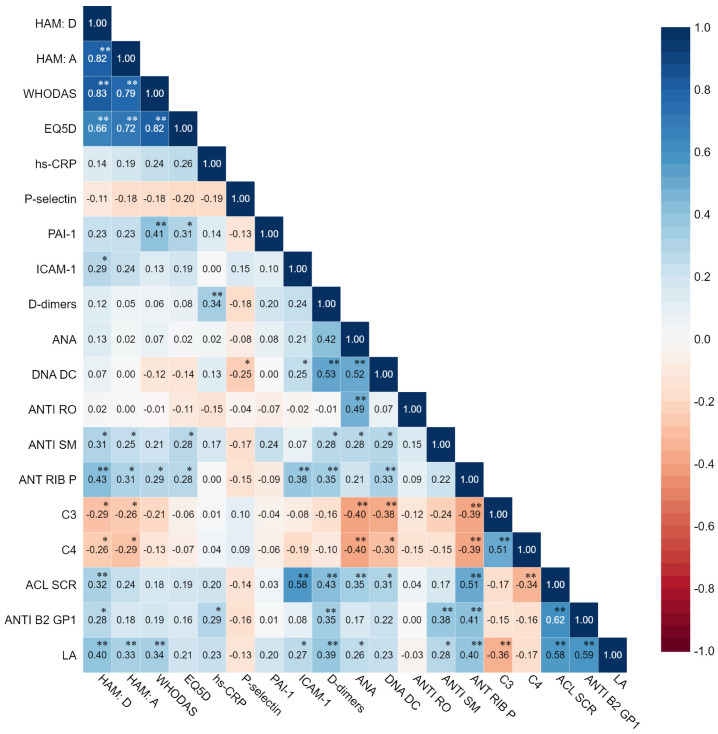
Correlation heatmap of biomarkers associated with thrombotic and inflammation pathways and depression, anxiety, disability and quality of life in SLE subjects (** *p* < 0.01, * *p* < 0.05).

Correlations were made between biomarkers related to thrombotic and inflammatory events and the presence of anxiety, depression, level of disability and quality of life in subjects with SLE. The results are presented in Figure 3.

Moderate correlations were identified for depression and LA, ACL SCR, ANTI RIB P, and ANTI SM. For anxiety, moderate correlations were detected with LA and ANTI RIB P. Regarding the level of disability (WHODAS), it was moderately correlated with increased LA and PAI 1. The quality of life measured with EQ5D showed moderated correlations with PAI-1.

To identify the associations of specific biomarkers linked to thrombotic and inflammation pathway and neuropsychiatric manifestations, alongside disability level and quality of life in SLE patients, we performed a linear regression analysis. The results are depicted in Table 3.

For hs CRP, P-selectin and C4 differences between the intervals (low, average, high), a two-way ANOVA was performed. No difference was found for CRP and P-selectin, while for C4 intervals, the results suggested a statistically significant interaction between depression (HAM D) and average C4 levels, with F (1,63) = 7.66, *p* = 0.007. Additionally, HAM A and C4 intervals suggested interaction by normal levels versus low levels of C4 with F (1,63) = 5.68 and *p* = 020. As regards C3 and C4 interval of positive paraclinical data, the results suggested that as lower the C3 and C4 values are, as higher is the risk of manifesting both depression and anxiety (*p* < 0.005).

For the nonparametric test performed for HAM A, HAM D, WHODAS and EQD5 depicted in Table 4, the results suggests that positive patients for Anti RIB P, LA, ACL and ANTI B2 GP 1 manifest depression and anxiety in SLE patients, significantly more compared to negative biomarkers. For the other biomarkers analyzed in this research, like Anti-SM, DNA DC, ICAM positive, ANA, and Anti-RO, no significant differences were identified neither for anxiety, depression, disability or quality of life between positive and negative participants.

Since only patients with values below the interval were identified for PAI, and only one patient was positive for P-selectin, no differences reported for the two biomarkers could be identified.

The results of age and time since disease comparison regarding anxiety, depression, disability and quality of life, the results of two-way ANOVA showed significant differences only regarding the quality of life suggesting it decrease with aging (subjects older than 65 years vs. 30 to 44 years) and disease progression (higher than 10 years of SLE onset). No significant differences were found between age and disease evolution regarding anxiety, depression or disability level.

## 4. Discussion

In our study, based on HAM D and HAM A results and correlations, the presence of depression and anxiety in SLE patients was 86.15% and 98.46%, respectively, higher than previously reported. Recent systematic reviews and meta-analyses suggest that the overall prevalence of depression and anxiety among SLE patients is 35%, respectively 25.8%, with ranges from 8.7% to 78.6% for depression and from 1.1% to 71.4% for anxiety, but the authors emphasize that the heterogeneity of the assessment’s scales used for these two majors neuropsychiatric SLE manifestations make challenging to identify a precise prevalence of anxiety and depression among SLE patients [9]. At the same time recent cross-sectional research results on SLE patients showed depression in 61.5% of patients and anxiety in 54.4% and suggested a cut-off value for SLEDAI of 8.5 for the increased risk of neuropsychiatric SLE manifestations [37]. This research indicates a strong correlation between depression symptoms and severe disability in social participation, interpersonal relationships and life abilities. This is similar to recent data that show that moderate depression is associated with pain severity, disease activity and low quality of life, but also with an increased level of disability [38,39].

Our research results suggest that biomarkers related to coagulation or thrombotic pathway, alongside inflammation-specific markers, are associated with depression and anxiety in SLE patients. The linear regression results suggest that depression is associated with and predicted by high levels of Anti-RIB P and PAI 1. In contrast anxiety was instead associated with increased serum levels of LA and low C4. Disability and quality of life seem to be influenced by increased PAI 1, Anti RIB P and CRP and low DNA DC values. Since PAI 1 is a molecule linked both with thrombosis and inflammatory process, and recent research suggests that disease activity scores increase with higher PAI-1 serum levels [40], our research results suggest an association with depression (by linear regression results and with low correlation value). While in our research sample, PAI-1 serum levels were not remarkably elevated, recent research suggests that PAI-1 is associated with depression [41], while other research suggests that PAI-1 presence in serum is associated with NPSLE in general [42].

Previous research results reported a mean of EQD5 for SLE patients of 0.72 and suggested a more sensitive assessment tool for SLE disease manifestation and sociodemographic factors [43]. In our research, the mean score for EQD5 was 1.57, which also correlated with anxiety and Anti RIB P serum presence. Recent research regarding EQ-5D-3L results suggests that it is a more helpful tool for anxiety or depression screening in community settings than in hospitals [44].

Considering that inflammation biomarkers were found in subjects with depression, and also, the extrinsic coagulation pathway was linked with depression [2,45], the data from our research results depict that both coagulation and inflammation-specific molecules like LA, Anti RIB P, ACL, C4, PAI 1 and hs CRP are linked with depression and anxiety.

ACL seems to have been substantially described in subjects with mental disorders, considering it is an acquired coagulation abnormality [46]. Besides, mental stress affects coagulation and people with recurrent depression are associated with an increased thrombotic risk [45]. Furthermore, in NPSLE patients, ACL antibodies can be found within a range between 10 and 14.5% and were associated with cognitive impairment, headache and altered consciousness [47]. While in our research, ACL antibodies seemed to be correlated with anxiety and depression, when multiple variables were considered for building a linear model through stepwise linear regression, it seems that other biomarkers have the potential of predictors (Anti RIB P and PAI 1 for depression and LA and C4 for anxiety). Previous research results also suggest that Anti RIB P autoantibodies are found in a range from 10 to 47% of SLE patients, and the molecules’ presence was strongly correlated with psychiatric manifestations [33]. Our study’s group comparison results of ACL positive and negative subjects suggest that anxiety and depression are significantly higher in the positive ACL SLE patients.

While in our paper, ANTI B2 GP1 was associated both with depression and anxiety and strongly correlated with ACL; recent research found that ANTI B2 GP1 serum values were higher in SLE patients than control groups and also linked this biomarker with coagulation complications [48]. Additionally, recent research suggests that ANTI B2 GP1 is linked with thrombotic events in SLE patients but also correlated with other antiphospholipid antibodies [49].

Low C4 serum levels were identified as a potential risk factor for neuropsychiatric manifestation in SLE patients [50]. In our study, low levels of C4 serum were correlated but also associated with anxiety.

From the linear regression and group comparison, Anti RIB P, LA and C4 can be further considered indicators of neuropsychiatric manifestations in SLE patients, especially regarding anxiety and depression. In our study group, low complement and positive serology for antiphospholipid syndrome correlated both with depression and anxiety presence in SLE in accordance with data published regarding NPSLE [51,52,53].

In order to obtain a more precise diagnosis and an overview of the systemic manifestations of Lupus, several tools and good interdisciplinary collaboration are needed. Although there is heterogeneity in the evaluation scales used to identify anxiety and depression, both in the cases of NPSLE patients and psychiatric patients in general [8], carefully validated and reliable evaluation scales should be used for both research and clinical practice of anxiety and depression disorders [54,55].

In addition to identifying the elements of mental health, it is necessary to investigate the neurological effects through clinical and imaging tests. However, the impact on the quality of life and the level of disability must be considered [56].

We must emphasize that our results are based on patients without an active scoring for the disease activity; therefore, further research in similar directions should be encompassed on subjects with SLE disease activity and/or initially diagnosed with NPSLE.

The main limitations of our research are related to the fact that the study was conducted on a small number of participants and the need of a control group. Additionally, we must mention that no neurological evaluation of the patients was performed; thus, issues related to possible demyelination (even considered a rare phenomenon), myelopathy, seizures, or movement disorders were not investigated or identified. Therefore, besides the clinical, immunological, serum and psychiatric investigations, neurological assessment should be encompassed in future research for a more precise framework regarding NPSLE. Furthermore, two essential control groups related to our research limitations and future studies should be considered. One potential research could compare coagulation biomarkers and inflammation in SLE patients without depression and anxiety, and a second one comparing the inflammation and thrombotic pathway of NPSLE subjects (with anxiety and depression) and patients with psychiatric problems without Lupus.

## 5. Conclusions

The high prevalence of depression and anxiety in our sample size of SLE subjects, alongside with positive biomarkers for inflammation and following a thrombotic pathway suggest an active screening for these symptoms and manifestations in this population. There is a strong positive correlation between depression, anxiety and antibodies associated with anti-cardiolipin and anti beta2 glycoprotein I antibodies, lupus anticoagulant, ICAM-1, low C4 a and Anti RIB P antibodies. These data suggest that both autoimmune/inflammatory pathway and ischemic/thrombotic pathway, could contribute to depression and anxiety as NPSLE manifestation. We can consider that NPSLE-specific markers need to be specified for an early diagnosis and treatment guidelines should be adjusted.

## Figures and Tables

**Table 1:** Normal range and unit measure for the analyzed biomarkers.

Biomarker	Units/Technique	Normal Range
Hs CRP	mg/L (ELISA)	1–3
Anti SM	U/mL (ELISA)	<15
ANA	Titer IFI (indirect immunofluorescence staining)	<1/80
DNA DC	U/mL (ELISA)	<10
ANTI B2 GP1	U/mL (ELISA)	<10
ACL SCR	U/mL (ELISA)	<10
LA	Ratio normal value APTT/test(coagulometric method) screening and confirmation test	<1.2
P-selectin	ng/mL(ELISA)	<100
PAI 1	ng/mL (ELISA)	16.7–32.1
ICAM 1	ng/mL (ELISA)	<100
C3	mg/dL (Turbidimetry)	90–180
C4	mg/dL (Turbidimetry)	10–40
Anti RIB P	U/mL (ELISA)	<10
D-dimers	ng/mL (ELISA)	0–400

**Table 2:** Patient’s characteristic (n = 65).

Characteristic	Mean ± SD/Percent
Age	51.48 ± 13.85
Years of LES diagnosis	12.55 ± 8.10
Gender	
Male	5/7.69%
Female	60/92.31%
Marital status	
Widower	6/9.23%
Married	37/56.92%
Unmarried	8/12.31%
Divorced	14/21.54%
Social status	
No occupation	6/9.23%
Employee	24/36.92%
Retired	35/53.85%

**Table 3:** Linear regression results of SLE association of depression, anxiety, quality of life and biomarkers.

Outcome	Association/R^2^	B (Confidence Interval)	*p*
HAM D	Anti RIB P/0.183	0.070 (0.33–010)	<0.001
	PAI 1/0.258	2.949 (0.60–5.30)	0.014
HAM A	LA/0.109	0.485 (0.13–0.83)	0.007
	C4/0.166	−0.026 (−0.05–0.001)	0.043
WHODAS	PAI 1/0.169	8.965 (3.97–13.96)	0.001
	Anti RIB P/0.274	0.109 (0.04–0.18)	0.004
	DNA DC/0.330	−0.052(−0.10–0.01)	0.028
	hs CRP/0.377	16.356(1.07–31.64)	0.036
EQD5	PAI 1/0.096	0.164 (0.04–0.29)	0.012
	Anti RIB P/0.192	0.003 (0–0)	0.009
	DNA DC/0.256	−0.001 (0–0)	0.025
	hs CRP/0.320	0.460 (0.07–0.85)	0.021

**Table 4:** Differences of anxiety, depression and quality of life based on the analyzed biomarkers.

Outcome	n/%	Ham A (Mean Rank)	*p*	HAM D (Mean Rank)	*p*	WHODAS (Mean Rank)	*p*	EQD5 (Mean Rank)	*p*
Anti RIB P	positive = 28	45.27	<0.001	46.18	<0.001	29.96	0.260	42.59	<0.001
	negative = 37	23.72		23.03		35.50		25.74	
LA	positive = 31	41.03	0.001	41.66	<0.001	34.10	0.655	38.63	0.021
	negative = 34	25.68		25.10		32.00		27.87	
ACL	positive = 36	38.33	0.011	38.94	0.005	31.86	0.588	34.35	0.518
	negative = 29	26.38		25.62		34.41		31.33	
ANTI B2 GP1	positive = 31	39.42	0.009	38.31	0.031	31.23	0.470	35.82	0.245
	negative = 34	27.15		28.16		34.62		30.43	

## Data Availability

Available on request.
